# Conceptualising changes to tobacco and alcohol policy as affecting a single interlinked system

**DOI:** 10.1186/s12889-020-10000-3

**Published:** 2021-01-04

**Authors:** Duncan Gillespie, Jenny Hatchard, Hazel Squires, Anna Gilmore, Alan Brennan

**Affiliations:** 1 School of Health and Related Research, The University of Sheffield, Regent Court, 30 Regent Street, Sheffield, S1 4DA UK; 2 UK Centre for Tobacco and Alcohol Studies, Nottingham, UK; 3 SPECTRUM Consortium, Edinburgh, UK; 4 Tobacco Control Research Group, Department for Health, University of Bath, Bath, UK

**Keywords:** Systems, Complexity, Commercial determinants, Public health, Interventions, Modelling

## Abstract

**Background:**

To support a move towards a coordinated non-communicable disease approach in public health policy, it is important to conceptualise changes to policy on tobacco and alcohol as affecting a single interlinked system. For health economic models to effectively inform policy, the first step in their development should be to develop a conceptual understanding of the system complexity that is likely to affect the outcomes of policy change. Our aim in this study was to support the development and interpretation of health economic models of the effects of changes to tobacco and alcohol policies by developing a conceptual understanding of the main components and mechanisms in the system that links policy change to outcomes.

**Methods:**

Our study was based on a workshop from which we captured data on participant discussions on the joint tobacco–alcohol policy system. To inform these discussions, we prepared with a literature review and a survey of participants. Participants were academics and policy professionals who work in the United Kingdom. Data were analysed thematically to produce a description of the main components and mechanisms within the system.

**Results:**

Of the people invited, 24 completed the survey (18 academic, 6 policy); 21 attended the workshop (16 academic, 5 policy). Our analysis identified eleven mechanisms through which individuals might modify the effects of a policy change, which include mechanisms that might lead to linked effects of policy change on tobacco and alcohol consumption. We identified ten mechanisms by which the tobacco and alcohol industries might modify the effects of policy changes, grouped into two categories: Reducing policy effectiveness; Enacting counter-measures. Finally, we identified eighteen research questions that indicate potential avenues for further work to understand the potential outcomes of policy change.

**Conclusions:**

Model development should carefully consider the ways in which individuals and the tobacco and alcohol industries might modify the effects of policy change, and the extent to which this results in an unequal societal distribution of outcomes. Modelled evidence should then be interpreted in the light of the conceptual understanding of the system that the modelling necessarily simplifies in order to predict the outcomes of policy change.

## Background

Life-long disease prevention is a complex and difficult task requiring strong public health systems, individuals who are empowered to promote their own health, and health-supporting environments. To support a move towards a coordinated non-communicable disease approach in public health policy, it is important to conceptualise changes to policy on tobacco and alcohol as affecting a single interlinked system, e.g., acknowledging that there are relationships between tobacco and alcohol in policy formation and the outcomes of policy change for consumers, the economy and society. This includes understanding how policy changes might interact with the commercial interests of the tobacco and alcohol industries [1–3]. Health economic models have a role to play by helping to inform policy decision-makers about the potential societal outcomes of their interventions. However, for such models to most effectively inform policy, the first step in their development should be to develop a conceptual understanding of the aspects of system complexity that are likely to affect the outcomes of policy change [4].

The genesis of this study was our aim to extend the Sheffield Alcohol Policy Model (SAPM) [5] to allow it to model the potential outcomes of changes to policy on alcohol and tobacco. SAPM was developed in the context of the United Kingdom (UK) in consultation with a range of alcohol policy stakeholders, which helped to define the relevant policy options to investigate and the outcomes of most interest [6]. Much of the impact of the modelling results generated by SAPM has come from helping policymakers and other policy actors to debate the competing values underpinning policy goals and to consider the trade-offs involved [7]. To develop our joint tobacco and alcohol policy model, we are following a framework for health economic model development developed by Squires et al. [4]. This framework advises first taking a systems approach to understand the relevant mechanisms that link a change in policy to its outcomes, and to develop this understanding in consultation with academic experts and policy stakeholders [8, 9]. The representation of the system produced then provides a guide for the subsequent development of a health economic model (which will inevitably involve simplifications of complexity, depending on the available data) and for how the results of the model should be interpreted.

Our aim in this study was to support the development and interpretation of health economic models of the effects of changes to tobacco and alcohol policies by developing a conceptual understanding of the main components and mechanisms in the system that links policy change to outcomes. We setup our investigation to answer the question, ‘How could we model the effects of policies that target tobacco and/or alcohol consumption in common terms?’ To answer, we applied a version of Problem Structuring Methodology to elicit a structured representation of the joint tobacco–alcohol policy system from the discussions of a set of UK academic and policy participants [10, 11].

## Methods

### Approach

Our study was based on a workshop from which we captured data on participant discussions. To inform these discussions, we prepared with a literature review and a survey of participants. We analysed the data collected to produce a summary of the main components of the system and the main mechanisms. Our results section presents the answer to our research question in five parts. (1) Why we should model the effects of policies that target tobacco and/or alcohol consumption in common terms. (2) The policy options we should consider. (3) The groups in society we should consider. (4) The main mechanisms that link policy change to outcomes. (5) What we still need to know.

### Preparatory work

We prepared with: a survey of participants to gauge their opinions and to obtain a starting-point in our understanding of the system; a scoping review of the academic and policy literature [12]. We summarised the findings of our survey and review and provided this information to participants at the start of our workshop (Additional file 1 describes the design and findings of our survey and review; Additional files 2 and 3 show the information that we provided to participants and facilitators at the start of our workshop).

#### Survey

We surveyed participants in July/August 2015. The survey contained four questions: What did participants feel that this exercise could produce that is of benefit?; What policy options did participants consider to be ‘good candidates’ to consider?; How might a policy-induced change in smoking affect drinking, and vice versa?; What should future collaborative research do to inform a coordinated policy strategy on tobacco and alcohol?

#### Review

We first selected seven documents that summarised UK and global research and policy and from them defined five discrete policy themes (Price, Place, Person, Promotion, Prescriptive) and one cross-cutting theme (Industry Regulation), further adapting the 4Ps marketing mix from McGill et al [13]. We then searched titles of English language articles and reviews in the Science Citation Index Expanded and Social Sciences Citation Index for literature published from 2005 to 2015 that referred to each policy theme and both tobacco and alcohol. We supplemented our findings with relevant literature cited in the papers found, and from the research team’s literature databases. We selected 25 research papers, which we used alongside the survey results to produce a brief description of each policy theme.

### Workshop

In our workshop, held in September 2015, participants were organised into five groups of 3–5 people with a mix of tobacco/alcohol expertise and academic/policy backgrounds. Each participant was provided with a notebook and told that the notes they made during the workshop would constitute a major part of our data. In the first session, participants were asked to ‘brainstorm’ one of the discrete policy themes (Price, Place, Person, Promotion, Prescriptive). Each group had a facilitator, who oriented discussion around the construction of a diagram showing the key components of the system and the mechanisms that link policy change to tobacco and/or alcohol consumption. In the second session, groups spent time understanding and critiquing other groups’ diagrams. As groups rotated around each other’s diagrams, the facilitator from each group remained by their own diagram to explain it to the other groups. Participants were also asked to comment on the potential evidence gaps and how these might be filled with existing or future data and research. Finally, in plenary, each diagram was presented, followed by a discussion of similarities and differences between policy themes, cross-cutting mechanisms, and priorities for future research. Discussions were recorded in the form of each group’s diagram and in the written notes made throughout the workshop by the facilitators and participants.

### Participants

We selected potential participants for research and policy experience related to tobacco and/or alcohol. We chose not to involve lay members of the public at this stage of model development because our aim was to elicit a broad overview of the system, but we will involve lay participants in future projects that are focused on a specific policy problem. We also did not involve members of the tobacco or alcohol industries in order to avoid conflicts of interest. The people we invited to participate were academics (from UK research networks) and policy professionals (from UK government agencies or non-governmental organisations involved in health advocacy). We invited an initial set of people by email, and then a further set based on suggestions by invited individuals.

### Analysis

The data comprised: the responses to our survey, the briefing information provided to participants at the start of our workshop; the facilitator and participant workshop notes; the diagrams from our workshop. We uploaded all data into the software NVivo10 [14]. The data were analysed by DG and JH. We initially indexed phrases, sentences or paragraphs by policy theme; data were indexed under more than one theme where relevant.

To identify the main components of the system and the main mechanisms, we indexed the data according to a framework from Soft Systems Methodology, which conceptualises a system as a set of interconnected elements [10, 15]. For this study, our interpretation of the elements of the system was as follows. *Customers,* are members of the public who might be affected by changes to tobacco or alcohol policy. We were particularly interested in understanding how individual characteristics (e.g. smoking or drinking habits, health, or socio-economic status) might modify how they respond to policy changes. *Actors,* are the people who perform the tasks in the system, but who have limited control over the system (e.g. health practitioners, retail workers, community groups or enforcement agencies). *Transformation,* describes the mechanisms that determine the outcomes of a policy change. *Worldview,* describes the objectives held within the system (e.g. the tobacco industry might wish to maximise profits) and the beliefs and values that underpin these. *Owners,* are the individuals or organisations who exert control over the system (e.g. government policymakers or corporate strategists). *Environment,* is the external factors which influence but do not control government deliberation among policy options.

We then identified further themes within the data and any references to evidence gaps or potential future research. To help identify themes we referred to five existing schema: individual access to products or services [16], marketing activities [17], corporate influences on policy [2], social theory on individual interactions with their environment [18, 19]. We also used the COM-B scheme to represent individual behavioural complexity [20], which we interpreted as: *Capability*, comprising factors such as knowledge and self-control; *Opportunity*, which describes the factors that prompt behaviours and might be part of either someone’s physical environment (e.g. neighbourhood characteristics) or their social environment (e.g. exposure to ideas); *Motivation*, which focuses on decision-making (e.g. reflective and automatic choices to consume tobacco or alcohol). *Behaviour*, which captures the details of tobacco and alcohol consumption. Themes were identified, defined and merged as necessary through discussion and agreement between JH and DG [21].

The result of our data analysis was a document containing a description of the components and mechanisms within the system (we have deposited this document in an online data repository [22]). We used it and the participant responses to our survey as references to inform the five part answer to our research question.

## Results

We initially invited 23 people (20 academic, 3 policy) and then 9 people from recommendations (4 academic, 5 policy). Of those invited, 24 completed the survey (18 academic, 6 policy); 21 attended the workshop (16 academic, 5 policy). To preserve anonymity, we identify participants by a unique number and attribute quotations to individuals in terms of whether they were from an academic or policy background. All quotations are taken from our survey.

### Why we should model the effects of policies that target tobacco and/or alcohol consumption in common terms

In our survey, participants identified the potential benefits to policymaking of being able to make fair comparisons between the effects of changes to tobacco policy and alcohol policy, and to understand how changes to tobacco policy and alcohol policy might combine to affect the same outcomes. For example, one participant commented.

“*I think it would be incredibly useful to have a tool to permit estimates of benefits of different tobacco and alcohol policies that were relatively comparable, and accounted for positive (or negative) synergies. Reaching that goal through expert discussion and possibly consensus would add weight and face validity to the tool.*”

(Participant 1, policy background)

Participants also highlighted that to understand the effects of policy changes on socio-economic or health inequalities, it is important to understand how changes to tobacco and alcohol policy might affect individuals differently, thinking particularly of the characteristics of people who both smoke and drink to harmful levels.

“*Given the numbers of people who use both tobacco and drink alcohol at levels that increases risk of health harm an understanding of the impact of policies would greatly aid policy development. Policies are often favoured that have a demonstrable impact on a given at-risk population. Both alcohol and tobacco harm place a burden on individuals, society and the public sector and in this time of reduced resources, demonstrating the impact of policies allows focus on best-buy policies.*”

(Participant 2, policy background)

Participants were also aware that taking part in this study was an opportunity for them to share expertise across the tobacco and alcohol fields, and to develop a better understanding of the ways in which combinations of policies might affect individuals.

“*In the tobacco and alcohol fields we tend to operate in substance specific research which, while acknowledging the links between these two behaviours and their determinants, rarely looks at the two issues together. This is particularly important when considering the impact of different policies, both intended and unintended consequences. This exercise could help us start to progress our understanding and therefore modelling in ways which may have important policy implications.*”

(Participant 3, academic background)

### The policy options we should consider

Table 1 presents a list of policy options that our data suggested are relevant to consider in the context of the joint tobacco–alcohol policy system. For example, participants discussed how specialist treatment services might be changed to better support people who both smoke and drink, e.g. whether smoking and drinking should be treated sequentially or simultaneously, and the feasibility, time requirements and costs of linking tobacco and alcohol treatment services. Participants’ discussions also highlighted the tobacco–alcohol differences in policy options due to: (*i*) The way that UK society perceives alcohol as less harmful to health and more beneficial to society and the economy than tobacco; (*ii*) The UK having stronger existing policy on tobacco than alcohol, which partly reflects adherence to international policy through the Framework Convention on Tobacco Control (FCTC) [24].

**Table 1:** Policy options to reduce tobacco and alcohol consumption. The six policy themes were defined based on the information from our review and survey. The examples of policy options within each theme are based on the information from our review, survey and workshop

Theme	Policy options
*Price* To reduce access to harmful products by raising the retail price and hence reducing affordability, whilst increasing the affordability of healthy options.	- Adjust the structure and rates of taxation. - Move to ‘fully specific’ taxation, i.e. all excise duty applied in proportion to the amount of harmful product content such as the concentration of ethanol in alcoholic beverages. - Introduce rules for the minimum amount of tax that must be applied to a product (c.f. minimum excise tax for tobacco). - Introduce minimum sales prices (cf. minimum unit pricing for alcohol). - Regulate multi-buy offers and discounts. - Introduce economic incentives for healthier products e.g. low tax for low alcohol beer.
*Place* To reduce access to harmful products, and encourage healthy options, by managing retailers and where consumption takes place.	- Increase penalties for retailers breaking the terms of licenses. - Restrict the number, density and location of retail outlets. - Restrict hours of sale. - Introduce licenses to sell tobacco and combine or coordinate licenses to sell tobacco and alcohol. - Raise the minimum age of sale and/or enforce current rules with proof of age initiatives and action to reduce proxy sales. - Regenerate neighbourhoods and town centres to create health-promoting environments. - Introduce smoke-free zones - Encourage alcohol-free social venues.
*Promotion* To inform people about the harms of consumption and promote healthy behaviours, whilst counteracting the strategies employed by the tobacco and alcohol industries to promote consumption.	- Target initiatives (e.g. social marketing or mass media campaigns) to specific groups of people to provide information on the health effects of consumption, and to promote and maintain healthy behaviours as the norm. - Support school-based programmes to improve mental well-being, resilience, self-control and social/personal competence skills that might help people resist influences to smoke or drink. - Design initiatives that combine health promotion messages across tobacco and alcohol e.g. by referring to common health harms such as cancer. - Design initiatives that raise awareness among the public and policymakers of the unhealthy effects of the companies that produce and market tobacco and alcohol products (cf. the US tobacco ‘Truth’ campaign [23]).
*Person* To strengthen the system of organisations and technology that encourages and supports people to quit or reduce consumption in the long term.	- Increase the funding and training of practitioners to deliver existing services. - Change procedures so that healthcare practitioners can identify people who both smoke and drink to harmful levels, and advise them why and how to reduce their smoking and drinking. - Change the structure of specialist services to better support people who both smoke and drink. - Support community groups that widen access to peer support and take the time to understand the context of a person’s life. - Provide flexible access to support for people with mental health problems at all levels of the continuum (in distress, acute or chronic).
*Prescriptive* To regulate the nature of and limit people’s exposure to tobacco and alcohol marketing, and in doing so to reduce the influence of that marketing on the culture of consumption.	- Prohibit marketing targeted to vulnerable people, e.g. people with mental health problems. - Regulate direct advertising e.g. the use of lifestyle messages and sponsorship of sports events. - Regulate indirect advertising e.g. third-parties talking about products on social media and product placement in films. - Regulate branding, packaging and sales quantity. - Regulate short-term sales promotion e.g. through branding and packaging, extra displays and other measures to stimulate publicity. - Regulate product content of harmful substances. - Regulate labelling so individuals understand contents, including warnings about health harms. - Bring marketing regulations for alcohol into alignment with regulations for tobacco under the Framework Convention on Tobacco Control (FCTC) [24].
*Industry regulation (cross-cutting)* To limit the ability for the tobacco and alcohol industries to influence the formation and effectiveness of public policy, and to recoup the public costs generated by tobacco and alcohol consumption.	- Limit the influence that tobacco and alcohol companies have on government policy, including direct lobbying and indirect influence through third party organisations and political donations. - Exclude psychoactive substances from trade agreements. - Bring regulations on access to and collaboration with government by the alcohol companies into alignment with tobacco companies under article 5.3 of the FCTC. - Engage with international organisations to regulate the activity of transnational tobacco and alcohol companies. - Monitor the responses of tobacco and alcohol companies to regulation. - Promote open and transparent reporting of tobacco and alcohol company activities e.g. marketing expenditure, lobbying activity and funding of third-party organisations. - Limit the influence that tobacco and alcohol companies have on the design and delivery of Person and Promotion initiatives. - Increase enforcement to minimise the trade in ‘illicit’ (i.e. tax free) products. - Introduce an annual levy on tobacco and alcohol companies, and hypothecate the money raised to pay for initiatives to reduce the societal costs of tobacco and alcohol consumption e.g. by funding healthcare, policing.

### The groups in society we should consider

**Fig. 1:**
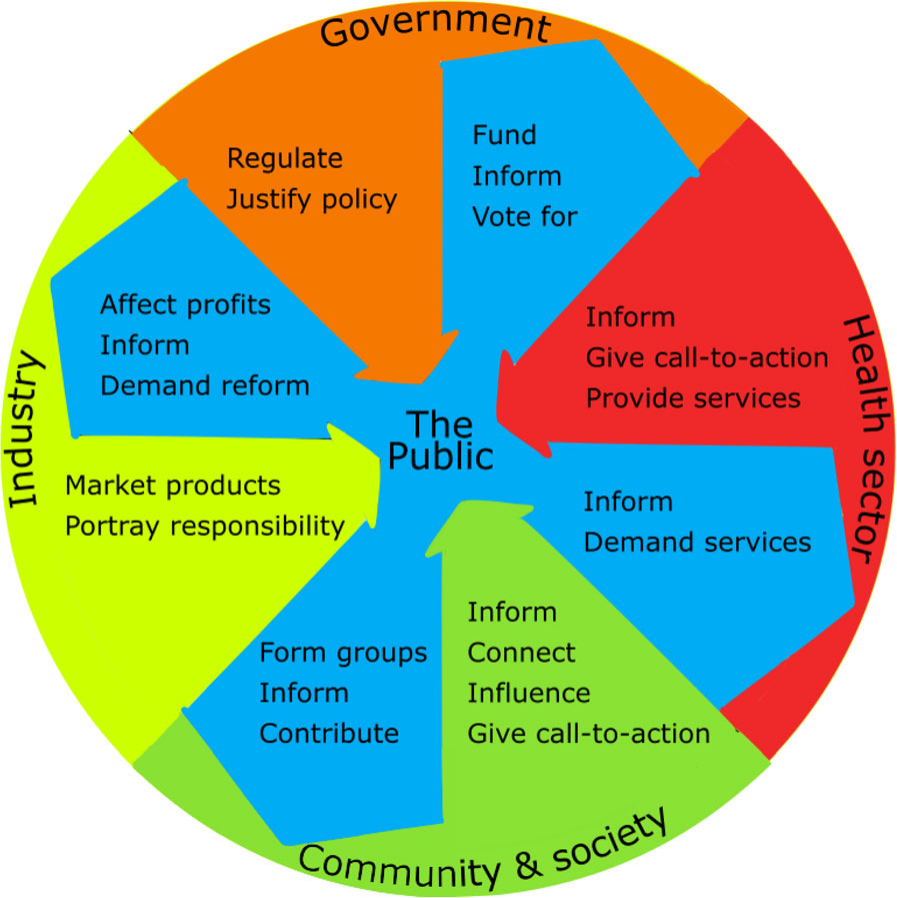
The groups in the joint tobacco–alcohol policy system, organised into a public-focused system. We derived the themes with which we have labelled the interactions from our data analysis

Our analysis identified five broad groups in society that influence the joint tobacco–alcohol policy system. *Government* – manages and regulates the system, with the crucial factor being that the values held by Government affect how the system is managed (e.g. the value placed on the economy vs. public health). *Industry* – manufactures, imports, markets and retails tobacco and alcohol products. *The Health Sector* – advocates for, informs, manages and delivers health-oriented activities. *Community & Society* – a diverse set of influential individuals and organisations (e.g. charities, think-tanks and media providers), who might advocate on behalf of, share objectives with or form partnerships with the other groups in the system. *The Public* – who we put at the centre of our representation of the system (Fig. 1). Members of the Public are subject to influences from each of the other four groups, with the main competition to influence the Public between Industry (who want to maintain their profits from product sales) and the Health Sector (who want to reduce unhealthy consumption). Industry and the Health Sector also compete to affect the Public indirectly by lobbying Government and forming partnerships with individuals and organisations in Community & Society. The Public feeds back into the system by informing, funding, influencing, and contributing to all of the other four groups.

### The main mechanisms that link policy change to outcomes

Our analysis identified two categories of mechanisms that modify the outcomes of policy changes: *Policy modification by individual behaviour* and *Policy modification by Industry*.

#### Policy modification by individual behaviour

**Fig. 2:**
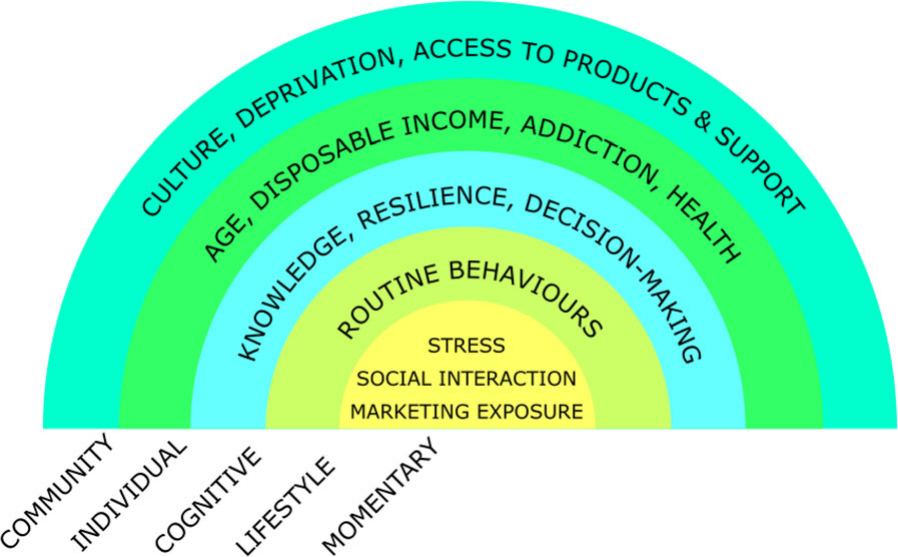
The determinants of tobacco and alcohol consumption behaviour. We identified the themes within this hierarchy from our data, guided by the schema cited in our methods

To support our understanding of how individuals might respond to policy change, we used our data to suggest a hierarchy of influences on tobacco and alcohol consumption behaviours (Fig. 2). To see how thinking of the effects of policy change in terms of a hierarchy of influences might be useful, consider our participants’ discussion of the effects of a policy that raises the price of alcohol in bars (affecting access to products in the community by changing affordability). Depending on an individual’s disposable income, they might decide to avoid the change by buying cheaper alcohol. This might in turn result in lifestyle changes to where they routinely socialise. On a particular social occasion, they might also compensate by binge-drinking cheaper supermarket alcohol with their friends before going to the bar (“pre-loading”), or by foregoing spending on cigarettes or a restaurant meal in order to afford being able to relax or socialise in a certain way by having a drink. Table 2 shows eleven mechanisms that we identified from our data through which individuals might modify the effects of a policy change. Each of these mechanisms could also be a reason why policy changes have linked effects on tobacco and alcohol consumption.

**Table 2:** Eleven mechanisms through which individuals might modify policy effects post-implementation. Our survey provided preliminary data on these mechanisms, focused on how changes to tobacco and/or alcohol policy might have linked effects on tobacco and alcohol consumption. The mechanisms in this table are based on our analysis of the data from our survey and workshop. We show the distribution of mechanisms across policy themes that was suggested by our data

Mechanism	Description	Price	Place	Promotion	Person	Prescriptive	Industry regulation
Maintain behaviour	Individuals do not change their consumption behaviour despite being exposed to the effect of the policy.	x	x	x	x	x	
Removal of constraints on other consumption	Individuals reduce consumption and in doing so increase their opportunity to consume other products e.g. by gaining disposable income or by moving drinking to locations where smoking is permitted.	x	x	x	x	x	
Replacement with other consumption	Individuals reduce consumption but replace it with increased consumption of other products e.g. people in recovery from alcohol or drug addiction might smoke more or eat sweets.	x	x	x	x	x	
Removal of triggers to consume other products	Individuals reduce consumption and in doing so disrupt the automatic relationship between behaviours e.g. drunkenness as a trigger for smoking.	x	x	x	x	x	
Amplify policy effects by social contagion	Individuals amplify policy effects by influencing the consumption behaviour of others (social contagion) e.g. by reducing the peer-pressure that others feel to consume in certain ways.	x	x	x	x	x	
Spatial avoidance of policy effects	Individuals change the source of their purchase or the location of their consumption e.g. relocating consumption to the home.	x	x			x	
Adapt by compensating	Individuals increase their intensity of consumption e.g. smoke ‘harder’ or ‘pre-load’ on alcohol before a night out.	x	x				
Change individual determinants of multiple behaviours	Individuals change multiple aspects of their behaviour because these behaviours are underpinned by a common aspect of individual variation that the policy has changed e.g. the motivation to be healthier or mental health problems.			x	x		
Fiscal avoidance of policy effects	Individuals ‘trade-down’ to a cheaper brand or source of purchase e.g. due to an increase in sales price.	x					
Adapt by foregoing	Individuals reduce their spending on other items to help maintain consumption e.g. prioritise spending on alcohol over food.	x					
Disruption of context for multiple behaviours	Individuals change multiple aspects of their behaviour because the policy has removed a key aspect of a multi-faceted context in which consumption normally occurs e.g. occasions for which smoking and drinking are integral.		x				

#### Policy modification by industry

We identified ten mechanisms by which the tobacco and alcohol industries might modify the effects of policy changes, grouped into two categories: Reducing policy effectiveness; Enacting counter-measures (Table 3). To illustrate how these mechanisms might operate, consider a Prescriptive policy change that restricts alcohol advertising to young people. To reduce effectiveness, alcohol corporations might choose not to comply with the restriction, or circumvent it by shifting advertising to less regulated channels (e.g. social media). Counter-measures might then be introduced such as making the affected products cheaper (e.g. by cross-subsidising or price promotions) or changing marketing effort to products that are not affected by the restriction. Next, consider an Industry Regulation policy change that aims to recoup costs to the Public generated by alcohol consumption (e.g. via a levy on corporate profits). This could be countered by alcohol corporations raising retail prices, with the knock-on effects that the Public might reduce their consumption, which would then reduce the tax revenue to Government. Corporate lobbying of Government and publicity might also seek to create doubt about the need for alcohol corporations to be subject to strong regulation (e.g. by questioning the evidence that their marketing increases harmful drinking), and to emphasise the cost of regulation due to the loss of the economic and social value of alcohol consumption. Participants talked about how Health Sector communications to Government and the Public could counter this, learning from past experience with tobacco corporations, by monitoring corporate political practices and clarifying the evidence on the potential health, economic and social outcomes of policies that prioritise public health. As one participant responded to our survey.

**Table 3:** Ten mechanisms through which Industry might modify policy effects. We initially identified a potential set of mechanisms from classifications of marketing activities [17] and tobacco industry influence on policy [2]. We used these potential mechanisms to inform our analysis of the data from our survey and workshop; based on these data, we described a final set of ten mechanisms. We show the distribution of mechanisms across policy themes that was suggested by our data

Strategy	Mechanism	Description	Price	Place	Promotion	Person	Prescriptive	Industry regulation
Reduce effectiveness	Non-compliance	Industry choose not to comply i.e. break the rules.					x	x
	Circumvention	Industry change to activities that are less regulated.					x	x
	Adaptation	Industry adapt to new regulation e.g. by finding loopholes.					x	x
	Avoidance	Industry avoid regulation i.e. moving out of the jurisdictional reach of regulations and continuing the same activities.					x	x
	Strategic cooperation	Industry cooperate with public health objectives but modify their delivery to still achieve some of their own objectives.			x	x	x	
Counter-measures	Pricing compensation	Industry cross-subsidise between the products whose pricing they control to weaken the impact of policies.	x				x	
	Passing-on costs to customers	Industry pass the costs of new policy onto customers via increases in sales prices, allowing maintenance of profits.	x	x				x
	Distribution compensation	Industry increase the range and availability of products to maintain their customer base.	x	x			x	
	Promotion compensation	Industry increase product promotion e.g. compensatory changes to sales quantity, packaging, advertising and publicity.	x		x		x	
	Information interference	Industry driven mass communication and publicity casts doubt on the evidence that consumption harms health.			x			x

“*Alcohol policy work in particular can learn a lot from tobacco in relation to industry responses – much of what needs to be done for alcohol has been done for tobacco and the industry reactions so far are very similar for both. Knowing how the industry will react and frame arguments against should, hopefully, save time.*”

(Participant 4, academic background)

### What we still need to know

We identified a set of eighteen research questions that indicate potential avenues for further work (Table 4). To maximise the usefulness of future research to policymaking, participants emphasised the importance of building networks of academics, policymakers and health policy advocates within a country, and internationally. There was also a general feeling that there was a need for greater knowledge exchange between tobacco and alcohol (but it was also noted that there is no reason to limit the focus to just tobacco and alcohol, and that future work might consider extensions to other fields, particularly the determinants of obesity).

**Table 4:** Eighteen research questions to better understand the effects of policy change. Initial ideas of research questions came from participants’ answers to our survey question “To better inform a coordinated policy strategy on tobacco and alcohol use, future collaborative research should...”. The questions in this table result from our analysis of data from our survey and workshop

**Price**	
1. Do price policies on tobacco and alcohol have regressive effects, what are the ethics of this issue and how could these effects be mitigated?	
2. How are price and behaviour across tobacco and alcohol purchase linked differently for youth and adult populations?	
3. How do the tobacco and alcohol industries differentiate products in the marketplace and how does this relate to the cross-price elasticity of demand for different products?	
4. How does demand for illicit drugs respond to tobacco and alcohol price rises?	
**Place**	
5. How can policies be better tailored to the needs of local populations, e.g. to urban vs. rural settings?	
6. What are the advantages and challenges of introducing licenses to sell tobacco and of linking them to licenses to sell alcohol?	
**Promotion**	
7. How are people’s perceptions of product harm affected by independent vs. industry-led health promotion messages?	
8. Would campaigns that combined health promotion messages across tobacco and alcohol produce stronger or weaker messages than focused substance-specific campaigns (e.g. alcohol: ‘Year of liver disease’; tobacco: ‘Stoptober’)?	
9. How can campaigns for tobacco be effectively transferred to alcohol e.g. the US tobacco ‘Truth’ campaign [23] to expose and counter industry tactics in marketing and information management?	
**Person**	
10. How can health professionals identify people who smoke and drink to harmful levels, and how can these people be supported in a feasible and cost-effective way?	
11. How can support to reduce smoking and drinking be made more accessible to people with mental health issues?	
12. How can community groups be helped to widen access to support to reduce smoking and drinking outside of the health service?	
**Prescriptive**	
13. What might be the health, societal and economic impacts of bringing marketing regulations for alcohol into alignment with the Framework Convention on Tobacco Control?	
14. How do the tobacco and alcohol industries use online marketing to circumvent regulations on advertising and how can regulation and policing be more effective?	
**Industry regulation**	
15. How can restrictions on industry corporate political activity be implemented effectively given:	
a) jurisdictional limitations over transnational companies?	
b) uncertainty regarding at which sector of industry measures should be targeted (e.g. producers, importers, retailers)?	
c) industry adaptation (e.g. third party lobbying, or gifts and hospitality to policymakers)?	
16. What are the marketing strategies employed to maintain consumer demand following new policies e.g. regulations on packaging or changes to tax?	
17. How do the tobacco and alcohol industries deflect responsibility for the harms of consumption from themselves to individual consumers?	
18. What is the potential for government to gain revenue from industry levies and use this to fund services to support people to reduce their consumption, or to pay for costs of consumption to society?	

## Discussion

Understanding the mechanisms that link a policy change to its outcomes is an essential first step in developing the structure of health economic models [4]. This understanding also provides a reference to aid the interpretation of the modelled estimates of the outcomes of policy change, given that there will often be relevant aspects of system complexity that cannot be included in a health economic model. This study attempted to take a broad overview of the joint tobacco–alcohol policy system, and our results show the main components and mechanisms that link tobacco and alcohol policy changes to their effects on smoking and drinking behaviour.

The existing evidence is largely focused on how individuals respond behaviourally to policy change. One example is how drinking behaviour responded to bans on smoking indoors in pubs and bars, for which there is evidence from England and Scotland of reductions in alcohol consumption by people who smoke [25, 26]. There is also evidence to suggest that if policy changes disrupt drinking contexts so that people more often drink where they cannot also smoke, then this could reduce the risk of relapse to smoking among former smokers [27]. In our workshop, one participant highlighted the concept of *critical health literacy* [28] as a useful way to understand individual variation in responses to policy change. This concept has three domains, which in the context of this study might be interpreted as: (1) how people seek, understand and critically appraise information on how tobacco and alcohol affect health; (2) how people understand their tobacco and/or alcohol consumption as being influenced by social factors (e.g. life stress or neighbourhood characteristics) and commercial factors (e.g. product advertising, price and availability); (3) the extent to which individuals participate in collective action to reduce the harmful societal impact of tobacco and alcohol consumption (e.g. by influencing their peers, organising community support groups, or voting for governments that prioritise public health).

A further body of evidence illustrates how the tobacco and alcohol industries might respond to policy change. For example, in response to tax increases, there is evidence that both the tobacco and alcohol industries decrease the pre-tax prices of cheaper products (helping to maintain their affordability) whilst increasing the pre-tax prices of more expensive products (for which a small additional rise in the retail price might be less noticeable to consumers) [29, 30]. In preparation for the ban on the sale of cigarettes with flavours such as menthol in the UK in 2020, the tobacco industry adapted by introducing new products that circumvented the regulation [31]. The new products included menthol accessories and cigarillos with menthol capsules, which look similar to conventional cigarettes and, in England and Wales, can be promoted with branded packaging at the point-of-sale. The market dominance of a few tobacco companies makes it easier for them to control prices and adapt their market strategies to reduce the effectiveness of and counter policy changes that aim to decrease tobacco consumption [32]. The alcohol industry is different in having a more diverse range of products and companies, and more price competition, but the tobacco and alcohol industries also share market strategies [3], and our results suggest that there are a common set of mechanisms by which they might modify the effects of policy change.

It is also important to consider the competition between Industry and the Health Sector to influence Government policymaking [2], which can affect whether policy is changed at all, and the details of the new policy design. In contrast to the tobacco industry, the alcohol industry is subject to less stringent forms of regulation, e.g. self-regulation and voluntary codes of practice, and continues to play a role in UK policymaking despite conflicts of interest [33]. Like the tobacco industry, the alcohol industry tries to shape its own regulatory environment by lobbying Government and influencing how the Public perceives the role of alcohol in society [3]. However, public policy formulation is a collective process, the result of which depends in part on how Industry and the Health Sector position their communications to Government and the Public, and respond to each other’s communications. The findings of health economic models form part of the Health Sector’s communications, providing independent evidence on the potential outcomes of policy change. The challenge for health economic modellers is to ensure that their evidence is valid and credible, and that it covers the full range of outcomes that Government are interested in, including the outcomes that feature in Industry communications.

The question then becomes, how to build a mathematical model of the mechanisms that link a change to tobacco and/or alcohol policy to its outcomes in the light of our understanding of the system? The final phase of Squires et al.’s [4] framework for model development is where the developers focus on a specific policy problem and consider the mechanisms that should be modelled and the data that might be used to inform these mechanisms. The framework advises that the developers document the simplifications and assumptions that they make so that these can be discussed with stakeholders and the feedback used to improve model validity and credibility. The advantage of using Squires et al.’s framework is that it draws on established problem structuring approaches that are used to support strategic decision-making in the face of large systems of uncertainties [10, 11]. Squires et al.’s framework was developed in response to the need to have a systematic approach to building model structures to inform decision-making when there was uncertainty about the model structure needed, the data that should be used to inform it, and the influence of the developers’ choices on the estimates of outcomes (see also the UK Government guidelines on the development of models to inform policy decisions [34]). The findings of the models that result are then fed back into the decision-making process, where it is essential for the interpretation of the modelled results to be informed by the prior conceptual understanding of system complexity.

Since the scope of our study spanned multiple policy themes, with limited time for discussion, our findings inevitably simplify a great deal of complexity and are therefore limited in how useful they are for informing models of specific policy problems. For example, participants were only able to have preliminary discussions about the potential outcomes of introducing licenses for retailers to sell tobacco, and the extent to which the terms of tobacco sales licenses should be consistent with the existing sales licenses for alcohol. Our findings were also influenced by how we designed the study, the individuals who participated, and the analysis methodology, all of which would need to be adapted to suit a particular policy problem. For example, our first investigation using the Sheffield Tobacco and Alcohol Policy Model aims to appraise the potential effects of changes to UK taxation on tobacco and alcohol. As the first step, we will develop a detailed understanding of the joint tobacco–alcohol tax policy system with relevant stakeholders, including lay members of the public. We will then use this understanding to guide our use of the available data to appraise the outcomes of a set of tax policy options that stakeholders consider relevant, in terms of the outcomes that they consider relevant.

In conclusion, model development should carefully consider the ways in which individuals and the tobacco and alcohol industries might modify the effects of policy change, and the extent to which this results in an unequal societal distribution of outcomes. Modelled evidence should then be interpreted in the light of the conceptual understanding of the system that the modelling necessarily simplifies in order to predict the outcomes of policy change.

## Supplementary Information

**Additional file 1.**

**Additional file 2.**

**Additional file 3.**

## Data Availability

The datasets generated during the study are available in the Figshare repository [22], DOI: 10.15131/shef.data.11861190
