# Supplementary material for: Conceptualising changes to tobacco and alcohol policy as affecting a single interlinked system
Source: BMC Public Health. 2021 Jan 4;21:17. doi: 10.1186/s12889-020-10000-3 (PMC7783976; doi:10.1186/s12889-020-10000-3)
Supplement: Supplementary file 1 — Additional file 1. [file 12889_2020_10000_MOESM1_ESM.pdf]

Appendix A – Pre-workshop research

1. SURVEY

Aims
We conducted an online survey of participants to gauge their opinions and to obtain a starting point to help set the background and direction for our subsequent workshop.

Methods
From discussions and piloting within the project team, we designed an online survey that took 10-20 minutes to complete. The survey began with a brief description of our study aims, followed by four questions:

1. To begin, please let us know what you feel that this exercise could produce that is of benefit.
2. Please name up to five alcohol and/or tobacco policy options that you consider to be 'good candidates' to consider jointly.
3. What cross-over[1] effects do you think might be most important to policy outcomes, and can you very briefly outline your understanding of how these might occur?
4. To better inform a coordinated policy strategy on tobacco and alcohol use, future collaborative research should....

The survey was emailed to participants in July/August 2015. Of 37 participants invited to participant in the study, 24 completed the survey.

Summary of responses

**Table A1:** Summary of responses to pre-workshop survey questions	
Q1: To begin, please let us know what you feel that this exercise could produce that is of benefit.	• Sharing of expertise and development of collaborations across the fields of alcohol and tobacco<br>• A step towards the potential benefits of a joint tobacco/alcohol policy model<br>• Better understanding of the ways in which policies can influence behaviours<br>• Identification of policies relevant to joint alcohol / tobacco modelling

[1] We defined cross-over effects as the effect of a policy-induced change in smoking on drinking, and vice versa. [secondary effect, knock-on effect]

	• Identification of relevant evidence and data (and gaps in these)<br>• Increase awareness of the need for action on alcohol
Q2. Please name up to five alcohol and/or tobacco policy options that you consider to be 'good candidates' to consider jointly.	• Industry regulation: illicit; policy influence; levy<br>• Person: general support; identification and brief advice; specialist services<br>• Place: age of sale; environment; outlet number/density; opening hours<br>• Prescriptive: restrict marketing; health warnings<br>• Price: minimum unit pricing; tax; general measures to reduce affordability<br>• Promotion: health effects; mass media and social marketing campaigns; healthy behaviour promotion
Q3. What cross-over effects do you think might be most important to policy outcomes, and can you very briefly outline your understanding of how these might occur?	• Changing disposable income<br>• Changing perceived affordability of products<br>• Changing volume of consumption<br>• Changing venue of consumption<br>• Changing co-occurrence of consumption<br>• Changing uptake and initiation<br>• Changing likelihood of quitting<br>• Changing likelihood of policy action<br>• Changing knowledge of risks
Q4. To better inform a coordinated policy strategy on tobacco and alcohol use, future collaborative research should...	• Engage in further data collection and new studies<br>• Entail modelling of joint or connected policy scenarios<br>• Focus on policy and research transfer between alcohol and tobacco<br>• Take an interdisciplinary approach<br>• Be strategic about engaging policy makers and other researchers in this research agenda

Use in the study

We analysed these survey responses after we had conducted our scoping review. We used the policy themes identified from the scoping review to structure the analysis of participants' suggestions of relevant policy themes. The survey responses informed the design of our participant workshop, and the policy briefs that we provided to participants in the workshop programme with the aim of providing a starting-point for discussions. The survey responses also formed part of the data used in our qualitative interpretive analysis.

2. REVIEW

Aims

We aimed to search the literature to provide preliminary information relating to our question 'How could we model the effects of policies that target tobacco and/or alcohol consumption in a common framework?' By scoping the literature, we hoped to identify and better understand relevant policies, or interventions, for tobacco and alcohol. This objective followed Arksey and O'Malley's [1] understanding of scoping reviews as an appropriate method to examine the extent, range and nature of research activity around a research question.

Methods

To understand the range of interventions relevant to consider for tobacco and alcohol, we first purposively selected seven policy documents to review (Table A2). We analysed their content by reducing them to text 'snippets' that referred to interventions, e.g., "increasing size of warning labels". We then organised these snippets into five intervention themes, partly based on the scheme used in McGill et al. [2]. We produced a summary definition of each theme and identified associated keywords. Based on the keywords, we designed a search string to find literature that referred to these interventions in the context of both tobacco *and* alcohol.

Table A2: Policy documents used to begin review of interventions.

**Document**	**Reason for selection**
Achieving world-class cancer outcomes: a strategy for England 2015–2020. Cancer Research UK, 2015.	UK policy options on alcohol and tobacco from the perspective of cancer prevention.
Health First: An evidence based alcohol strategy for the UK. Stirling University, 2013.	UK policy options for alcohol.
Smoking Still Kills: Protecting children, reducing inequalities. Action on Smoking and Health (ASH), 2015.	UK policy options for tobacco.
Global strategy to reduce the harmful use of alcohol. World Health Organisation (WHO), 2010.	Global policy options for alcohol.
Tackling alcohol-related harms: What policy approaches? Chapter 4 in Tackling Harmful Alcohol use: Economics and Public Health Policy. Organisation for Economic Co-operation and Development (OECD), 2015.	Global policy options for alcohol.
(MPOWER). World Health Organisation (WHO), 2013.	Global policy options for tobacco.
UK Centre for Tobacco and Alcohol Studies (UKCTAS) briefing pack of ongoing research. Prepared for the UKCTAS Strategic General Meeting, York. April 2015.	Summary of research within the collaborating centre.

We used these search strings to find literature dates 2055-2015 that referred to interventions in the context of both tobacco and alcohol we searched the titles of English language articles and reviews in the Science Citation Index Expanded and Social Sciences Citation Index. We used search strings that began with the synonyms for tobacco and alcohol and ended with the keywords for each intervention theme:

(tobac* OR cigar* OR nicotine OR smok*) AND (alcohol* OR ethanol OR drink*) AND (theme specific keywords)

We supplemented our reviews with any relevant literature cited in the included papers and the research team's own knowledge of the literature.

Review findings

Through our search of papers published up to 2015 (prior to our workshop), we identified 25 relevant papers, which we used to provide examples of the links between tobacco and alcohol under each of our policy themes (Table A3). An additional theme emerged from our scoping review: all the interventions identified could be affected by interventions directed at industry practices e.g. government regulation designed to limit industry interference with policymaking. We labelled this cross-cutting theme 'industry regulation'.

Table A3: Results of the literature search

Theme	Links between tobacco and alcohol
Promotion	School-based programmes aim to improve mental well-being, resilience, self-control and social/personal competence skills. The effects can be to:<br>● Lower tobacco and alcohol use among young people [3]<br>● Help them refuse offers, resist influences, correct misperceptions that use is normative [4].<br>● Make them less susceptible to influence by tobacco/alcohol advertising [5].
Person	● Brief alcohol interventions do not reduce smoking [6]. In non-dependent drinkers, changes in smoking were not found to be associated with changes in drinking [7].<br>● Smoking cessation treatment that incorporates brief alcohol intervention has been found to reduce alcohol use [8], but not in alcohol dependent smokers [9].<br>● In alcohol dependent individuals smoking abstinence reduced drinking [10]; smoking mildly increased relapse to drinking [11]; but nicotine can help with alcohol withdrawal symptoms [12].<br>● Alcohol use increased the risk of relapse to smoking, but alcohol use reduced when smoking stopped and remained lower for six months post-cessation [13].
Prescriptive	● In nicotine dependent, non-alcoholic smokers, imagery of social drinking was found to strengthen both cigarette and alcohol cravings. The same was found for imagery of smoking [14].<br>● Tobacco brand placement in the U.S. was limited in 1998, but this had little effect on (industry self-regulated) alcohol brand appearances, which tended to increase in youth-rated movies [15].<br>● Ads might link cigarette and alcohol sales, e.g., cigarette promotions featuring alcohol discounts or encouraging alcohol use [16].<br>● Preventing coalitions or co-ownership of tobacco, alcohol and advertising companies might prevent the industries sharing resources and lobbying power (see cross-cutting theme on industry regulation).<br>● Prevent alcohol/tobacco industry sponsorship of intergovernmental events, funding of educational initiatives, research, publications and sponsoring sporting and cultural events (see cross-cutting theme on industry regulation)..
Price	● In the U.S., increased cigarette tax was linked to reduced alcohol consumption and binge drinking among smokers [17]. But only in male smokers. Stronger effect for hazardous drinkers, young adults, and adults with low income.<br>● Reductions in alcohol consumption associated with increased cigarette tax were seen for beer and spirits but not wine [18].<br>● Increased cigarette price raised binge drinking at young ages, and raised heavy drinking among young females. It reduced binge drinking among African Americans and Hispanics, and heavy drinking among Hispanics [19].
Place	● Alcohol and tobacco use increased for individuals closer to alcohol and tobacco retailers [20].

	● In the U.S., smoke-free airs laws decreased consumption of beer and spirits but not wine [18]. Bans in restaurants and bars had more effect on beer and spirits, but increased demand for wine [21].<br>● In an international study, smoke-free policies caused small reductions in alcohol consumption by hazardous drinkers and in the frequency of alcohol consumption among heavy smokers [22].<br>● In England, smokers drank more than non-smokers before and after smoke-free policy [23]. The ban decreased drinking for smokers but increased drinking for non-smokers.<br>● In Scotland, smoke-free legislation was associated with reduced drinking behaviour in pubs and bars among moderate and heavy drinking smokers [24]. These moderate and heavy drinkers also reduced their pub attendance. But the smoke-free law did not increase drinking in the home.
Cross-cutting theme: Industry regulation	Sources [25-27]:<br>● Public health policy sectors can learn from each other, implementing policy more quickly and better.<br>● There is no current tobacco Responsibility Deal, so there is a divide between the respectability of the tobacco and alcohol industries.<br>● Transnational corporations employ common strategies, so it is likely that common rules for all industries would affect both tobacco and alcohol use. (including strategies to avoid government regulation)<br>● The tobacco industry is subject to stronger regulation that the alcohol industry - can the measures or perceptions applied to the tobacco industry also be applied to the alcohol industry?<br>● Countering the message by industry that policy should focus on heavy drinkers or ineffective individually-targeted information and educational approaches might create more political will for population-level policies that have the potential to affect both drinking and smoking.<br>● Disclosure of industry funding to e.g., alliances of trade unions, employees, and groups representing minorities opposing control policies.

Use in the study

The results of the review were combined with participants' responses to our survey to construct one-page outlines of each policy theme (with examples of interventions and bullet-point summaries of our survey and scoping review) (Appendix B). The briefs were included in the workshop programme to use as a basis for discussion. The results of our survey and review formed part of our data for subsequent thematic analysis.

References

1. Arksey H, O'Malley L. Scoping studies: towards a methodological framework. International journal of social research methodology. 2005;8(1):19-32.
2. McGill R, Anwar E, Orton L, Bromley H, Lloyd-Williams F, O'Flaherty M, et al. Are interventions to promote healthy eating equally effective for all? Systematic review of socioeconomic inequalities in impact. BMC Public Health. 2015;15(1):457. doi: 10.1186/s12889-015-1781-7.
3. Hodder RK, Daly J, Freund M, Bowman J, Hazell T, Wiggers J. A school-based resilience intervention to decrease tobacco, alcohol and marijuana use in high school students. Bmc Public Health. 2011;11. doi: 10.1186/1471-2458-11-722. PubMed PMID: WOS:000296388200002.
4. Botvin GJ, Griffin KW. School-based programmes to prevent alcohol, tobacco and other drug use. International Review of Psychiatry. 2007;19(6):607-15. doi: 10.1080/09540260701797753. PubMed PMID: WOS:000252535200003.
5. Wills TA, Gibbons FX, Sargent JD, Gerrard M, Lee H-R, Dal Cin S. Good Self-Control Moderates the Effect of Mass Media on Adolescent Tobacco and Alcohol Use: Tests With Studies of Children and Adolescents. Health Psychology. 2010;29(5):539-49. doi: 10.1037/a0020818. PubMed PMID: WOS:000282116000011.
6. McCambridge J, Jenkins RJ. Do brief interventions which target alcohol consumption also reduce cigarette smoking? Systematic review and meta-analysis. Drug and Alcohol Dependence. 2008;96(3):263-70. doi: 10.1016/j.drugalcdep.2008.03.011. PubMed PMID: WOS:000257604700008.
7. Kahler CW, Borland R, Hyland A, McKee SA, O'Connor RJ, Fong GT, et al. Quitting smoking and change in alcohol consumption in the International Tobacco Control (ITC) Four Country Survey. Drug and alcohol dependence. 2010;110(1):101-7.
8. Kahler CW, Metrik J, LaChance HR, Ramsey SE, Abrams DB, Monti PM, et al. Addressing Heavy Drinking in Smoking Cessation Treatment: A Randomized Clinical Trial. Journal of Consulting and Clinical Psychology. 2008;76(5):852-62. doi: 10.1037/a0012717. PubMed PMID: WOS:000260070300014.
9. Cooney NL, Litt MD, Cooney JL, Pilkey DT, Steinberg HR. Concurrent brief versus intensive smoking intervention during alcohol dependence treatment. Psychology of Addictive Behaviors. 2007;21(4):570-5. doi: 10.1037/0893-164x.21.4.570. PubMed PMID: WOS:000251356100016.
10. Cooney NL, Litt MD, Sevarino KA, Levy L, Kranitz LS, Sackler H, et al. Concurrent Alcohol and Tobacco Treatment: Effect on Daily Process Measures of Alcohol Relapse Risk. Journal of Consulting and Clinical Psychology. 2015;83(2):346-58. doi: 10.1037/a0038633. PubMed PMID: WOS:000351945200010.
11. Dawson DA, Goldstein RB, Grant BF. Rates and Correlates of Relapse Among Individuals in Remission From DSM-IV Alcohol Dependence: A 3-Year Follow-Up. Alcoholism: Clinical and Experimental Research. 2007;31(12):2036-45.
12. Gulliver SB, Kamholz BW, Helstrom AW. Smoking cessation and alcohol abstinence: What do the data tell us? Alcohol Research & Health. 2006;29(3):208-13.
13. Kalman D, Kim S, DiGirolamo G, Smelson D, Ziedonis D. Addressing tobacco use disorder in smokers in early remission from alcohol dependence: The case for integrating smoking cessation services in substance use disorder treatment programs. Clinical Psychology Review. 2010;30(1):12-24. doi: 10.1016/j.cpr.2009.08.009. PubMed PMID: WOS:000273830100002.
14. Erblich J, Montgomery GH, Bovbjerg DH. Script-guided imagery of social drinking induces both alcohol and cigarette craving in a sample of nicotine-dependent smokers. Addictive Behaviors. 2009;34(2):164-70. doi: 10.1016/j.addbeh.2008.10.007. PubMed PMID: WOS:000262197200005.
15. Bergamini E, Demidenko E, Sargent JD. Trends in Tobacco and Alcohol Brand Placements in Popular US Movies, 1996 Through 2009. Jama Pediatrics. 2013;167(7):634-9. doi: 10.1001/jamapediatrics.2013.393. PubMed PMID: WOS:000323550200013.
16. Jiang N, Ling PM. Reinforcement of Smoking and Drinking: Tobacco Marketing Strategies Linked With Alcohol in the United States. American Journal of Public Health. 2011;101(10):1942-54. doi: 10.2105/ajph.2011.300157. PubMed PMID: WOS:000295657400034.
17. Young-Wolff KC, Kasza KA, Hyland AJ, McKee SA. Increased Cigarette Tax is Associated with Reductions in Alcohol Consumption in a Longitudinal US Sample. Alcoholism-Clinical and Experimental Research. 2014;38(1):241-8. doi: 10.1111/acer.12226. PubMed PMID: WOS:000329885900029.
18. Krauss MJ, Cavazos-Rehg PA, Plunk AD, Bierut LJ, Grucza RA. Effects of State Cigarette Excise Taxes and Smoke-Free Air Policies on State Per Capita Alcohol Consumption in the United States, 1980 to 2009. Alcoholism: Clinical and Experimental Research. 2014;38(10):2630-8.

19. McLellan DL, Hodgkin D, Fagan P, Reif S, Horgan CM. Unintended consequences of cigarette price changes for alcohol drinking behaviors across age groups: evidence from pooled cross sections. Substance Abuse Treatment Prevention and Policy. 2012;7. doi: 10.1186/1747-597x-7-28. PubMed PMID: WOS:000308906500001.
20. Weitzman ER, Chen YY, Subramanian SV. Youth smoking risk and community patterns of alcohol availability and control: a national multilevel study. Journal of Epidemiology and Community Health. 2005;59(12):1065-71. doi: 10.1136/jech.2005.033183. PubMed PMID: WOS:000233271200014.
21. Gallet CA, Eastman HS. The impact of smoking bans on alcohol demand. Social Science Journal. 2007;44(4):664-76. doi: 10.1016/j.soscij.2007.10.008. PubMed PMID: WOS:000252327700006.
22. Kasza KA, McKee SA, Rivard C, Hyland AJ. Smoke-free bar policies and smokers' alcohol consumption: findings from the International Tobacco Control Four Country Survey. Drug and alcohol dependence. 2012;126(1):240-5.
23. Orbell S, Lidierth P, Henderson CJ, Geeraert N, Uller C, Uskul AK, et al. Social-Cognitive Beliefs, Alcohol, and Tobacco Use: A Prospective Community Study of Change Following a Ban on Smoking in Public Places. Health Psychology. 2009;28(6):753-61. doi: 10.1037/a0016943. PubMed PMID: WOS:000271817400013.
24. McKee SA, Higbee C, O'Malley S, Hassan L, Borland R, Cummings KM, et al. Longitudinal evaluation of smoke-free Scotland on pub and home drinking behavior: Findings from the International Tobacco Control Policy Evaluation Project. Nicotine & Tobacco Research. 2009;11(6):619-26. doi: 10.1093/ntr/ntp020. PubMed PMID: WOS:000266698600007.
25. Moodie R, Stuckler D, Monteiro C, Sheron N, Neal B, Thamarangsi T, et al. Profits and pandemics: prevention of harmful effects of tobacco, alcohol, and ultra-processed food and drink industries. The Lancet. 2013;381(9867):670-9.
26. Jiang N, Ling P. Vested interests in addiction research and policy. Alliance between tobacco and alcohol industries to shape public policy. Addiction. 2013;108(5):852-64.
27. Casswell S. Vested interests in addiction research and policy. Why do we not see the corporate interests of the alcohol industry as clearly as we see those of the tobacco industry? Addiction. 2013;108(4):680-5.
