# Supplementary material for: Conceptualising changes to tobacco and alcohol policy as affecting a single interlinked system
Source: BMC Public Health. 2021 Jan 4;21:17. doi: 10.1186/s12889-020-10000-3 (PMC7783976; doi:10.1186/s12889-020-10000-3)
Supplement: Supplementary file 2 — Additional file 2. [file 12889_2020_10000_MOESM2_ESM.pdf]

# Tobacco and Alcohol Policy Modelling - developing a common policy modelling framework

## Workshop Programme

| Organisers | |
|---|---|
| Dr Duncan Gillespie | duncan.gillespie@sheffield.ac.uk |
| Dr Jenny Hatchard | j.hatchard@bath.ac.uk |

| Logistics | |
|---|---|
| **Place** | The Institute of Alcohol Studies, Alliance House, 12 Caxton Street, London SW1H 0QS. Click here for a map. The nearest tube stop is St James' Park (Circle & District lines). |
| **Date & Time** | 8th September 11am to 4pm (lunch included). |
| **Travel reimbursement** | Please bring your receipts to the workshop and Duncan will give you the appropriate expenses form. |

| Ethics & consent | |
|---|---|
| **Ethics approval** | Ethical approval for this project was obtained from the School of Health and Related Research Ethics Committee at the University of Sheffield, UK. |
| **Information sheet** | Please see the copy of the project information sheet in your welcome packs. |
| **Consent** | We will ask all participants who have not yet completed a consent form to do so at the workshop. |

| Note taking |
|---|
| **YOUR NOTES ARE OUR DATA!** We will give you notebooks to record any comments that you make during the day AND any unspoken thoughts that you think might be relevant. |

| Timetable | | |
|---|---|---|
| **Time** | **Session** | **Description** |
| 11:00 to 11:30 | **Introduction** | We will introduce each other, how the day will run and the responses to the pre-workshop survey. |
| 11:30 to 13:00 | **Session 1: Brainstorming** | Participants will form groups of 3-5 plus a facilitator.<br>Each group will focus on a different policy area (**see Policy Briefs appendix**).<br>Begin with a short discussion of how to proceed (**see Logic Model Instructions appendix**).<br>Then on flipchart paper begin to construct your logic model - **discussing and making notes as you go.** |
| 13:00 to 13:45 | **Lunch** | Each group's logic model will be displayed around the room. |
| 13:45 to 15:00 | **Session 2: Consolidation** | In groups rotate around logic models to critique and discuss the available evidence.<br>The facilitator from each group will remain by their own logic model to explain it to others. |
| 15:00 to 15:05 | **Short break** | |
| 15:05 to 16:00 | **Plenary session** | The facilitators will briefly summarise the comments received on each logic model.<br>We will then have an open discussion (thinking across all the logic models) of:<br>- What elements future policy assessments should focus on.<br>- The needs for future research. |
| Post-workshop | **Comments on our analysis of the outcomes** | We will analyse the notes and logic models and present the results to all participants for feedback. |

## Definitions

**Smoking:** The frequency, quantity and context of tobacco smoke inhalation; the action or habit of inhaling tobacco smoke.

**Drinking:** The frequency, quantity and context of alcohol intake; the action or habit of consuming alcoholic drinks.

**Cross-over effect:** The effect of a policy-induced change in smoking on drinking, and vice versa. [secondary effect, knock-on effect]

**System:** A set of things working together as parts of a mechanism or an interconnecting network; a complex whole. [Our focus is the mechanisms that link the implementation of tobacco and alcohol policies to outcomes in terms of smoking and drinking.]

**Model:** A simplified description, especially a mathematical one, of a system, to assist calculations and predictions; a representation of judgements about the relationship between model inputs and outputs. [Our ultimate goal is to construct a model of tobacco and alcohol policies to assess the effects on smoking and drinking.]

**Logic model:** A basic structure of a system; the key factors, concepts or variables and the presumed relationship among them. [Our aim is to develop logic models for a set of policy options relevant to smoking and drinking.]

## How we arrived at this workshop

- Duncan's perspective

In 2014, we started the task of expanding the Sheffield Alcohol Policy Model to include smoking as well as drinking behaviour. This was part of our commitment to the UK Centre for Tobacco and Alcohol Studies (UKCTAS) to develop health economics modelling for tobacco and alcohol.

The most important thing is to capture the "jointness" of the two behaviours. I am still coming to realise how complicated capturing "jointness" actually is. But I do understand that to build a joint alcohol and tobacco policy model, we need to understand the links at various levels, starting with policy implementation and effects on behaviour.

So what is the point of doing this?

We need a joint model to incorporate the effects that changes caused by alcohol policy have on tobacco consumption, and vice versa. We are also taking a first step to supporting a coordinated policy strategy that covers a range of risk factors, tobacco, alcohol, diet, exercise etc.

But also…
Tobacco and alcohol researchers could learn from each other. We need more cross-talk on science and policy between the fields of tobacco and alcohol. We're hoping to facilitate this and bring greater coordination.

The final point is inequalities, or the equity of policy effects / the distribution of policy effects across society. We will be writing down the "mechanisms of effect" of different policy options and those mechanisms will contain elements, e.g., differences in consumption or health awareness, that mediate inequalities.

Therefore, what we are aiming for is a set of logic models that capture the "jointness" in policy effects and show how inequalities arise (see Logic Model Instructions appendix). These will tackle five policy areas identified from the scoping review and pre-workshop survey (see Policy Briefs appendix).

When we reflect on these logic models, we will hopefully draw out where existing data can be applied, and where future research is needed.

- Duncan

# Logic Model Instructions Appendix

## WHAT we're aiming for – an example logic model

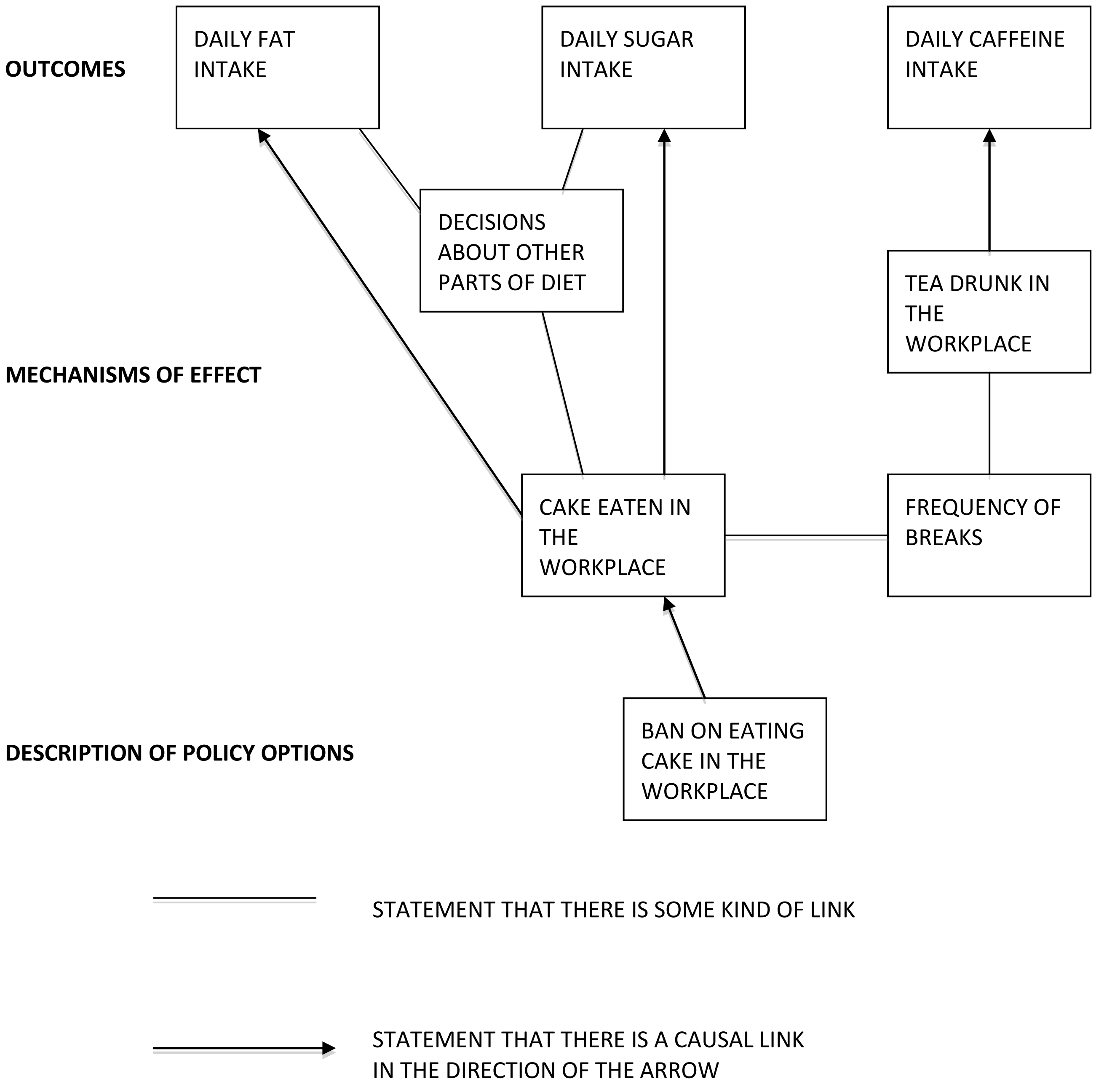


| Optional ways to add detail to a link | |
|---|---|
| **S** STRONG LINK | **+** WHEN ONE SIDE OF THE LINK INCREASES, SO DOES THE OTHER SIDE |
| **W** WEAK LINK | **–** WHEN ONE SIDE OF THE LINK INCREASES, THE OTHER SIDE DECREASES |

## HOW we'll get there

**PARTICIPANTS**

↓

**FORM 5 GROUPS**

- Each group has a different policy area
- Groups should be a mix of tobacco/alcohol, research/policy expertise

↓

**READ THE POLICY BRIEF**

- Identify a specific policy option as your initial focus
- Try to reflect what is happening or might happen in the UK

↓

**DISCUSS AND MAKE A LIST OF**

- OUTCOMES for smoking and drinking
- AGENTS in the system, e.g., industry, government, individuals
- FACTORS, CONCEPTS, VARIABLES that you want to include, e.g., from models of behaviour

↓

**BEGIN TO DRAW YOUR LOGIC MODEL**

- Do you need to represent subgroups? E.g., smokers/non-smokers, age, sex, health, income
- What are the similarities and differences in the mechanisms of effect for tobacco and alcohol policy?
- How might one policy option affect both smoking and drinking?

# Policy Briefs Appendix

**(you just need to focus on the policy area that you choose in the workshop)**

# Methods

1. **Scoping review**
   a. Policy documents: we reviewed a set of policy focused documents (Table 1) to produce a list of policy options relevant to tobacco and alcohol.

   b. Literature search: we searched articles and reviews with tobacco and alcohol in the title, plus keywords on policy from 1a.

2. **Pre-workshop survey**
   a. What are the policy options relevant to tobacco and alcohol?

   b. How might implementing a policy affect tobacco AND alcohol?

3. **Synthesis & summary**
   a. Identification of 5 policy areas. These are not mutually exclusive and were chosen to cover interesting areas for 'systems thinking'.

   b. List examples within each policy area.

   c. Summarise the links between tobacco and alcohol.

**Table 1.** Sources for scoping review of policy options.

| Document | Reason for selection |
|---|---|
| Achieving world-class cancer outcomes: a strategy for England 2015–2020. Cancer Research UK, 2015. | UK policy options on alcohol and tobacco from the perspective of cancer prevention. |
| Health First: An evidence based alcohol strategy for the UK. Stirling University, 2013. | UK policy options for alcohol. |
| Smoking Still Kills: Protecting children, reducing inequalities. Action on Smoking and Health (ASH), 2015. | UK policy options for tobacco. |
| Global strategy to reduce the harmful use of alcohol. World Health Organisation (WHO), 2010. | International policy options for alcohol. |
| Tackling alcohol-related harms: What policy approaches? Chapter 4 in Tackling Harmful Alcohol use: Economics and Public Health Policy. Organisation for Economic Co-operation and Development (OECD), 2015. | International policy options for alcohol. |
| Report on the global tobacco epidemic (MPOWER). World Health Organisation (WHO), 2013. | International policy options for tobacco. |
| UK Centre for Tobacco and Alcohol Studies (UKCTAS) briefing pack of ongoing research. Prepared for the UKCTAS Strategic General Meeting, York. April 2015. | Summary of research within the collaborating centre. |

# The policy briefs

We summarised our findings into five policy areas to investigate further at the workshop (Table 2).

**Table 2.** Five policy areas identified from the scoping review and pre-workshop survey. **Challenge questions are to stimulate thought and are not requirements.**

| | |
|---|---|
| **Promotion** | Social marketing, e.g., mass media public information campaigns.<br><br>**Challenge question:** Does your logic model capture how health promotion / education activities might counter industry advertising / marketing? |
| **Person** | Measures to help individuals reduce use, including:<br>• identification and brief advice<br>• general support and information for those interested in quitting/reducing<br>• specialist treatment services for those needing help to quit/reduce<br>• increased support for mental health issues<br><br>**Challenge question:** Using your logic model, could you describe how individual-based activities affect smoking and drinking for individuals with different levels of use and dependency? |
| **Prescriptive** | Regulation of industry advertising, marketing and sales, including:<br>• packaging and labelling recommendations or mandated requirements<br>• limiting the exposure of young people<br><br>**Challenge question:** Does your logic model show how industry, retailers and individuals are actors in the system that links specific policy options to smoking and drinking? |
| **Price** | Fiscal policies, including:<br>• taxation, e.g., excise duty, taxation based on product value or volume, VAT<br>• minimum unit pricing<br>• industry levies /fines (polluter pays)<br>• economic incentives (e.g., low tax for low alcohol beer)<br><br>**Challenge question:** Can your logic model show effects on smoking and drinking that act via individual / corporate budgets? |
| **Place** | Restrictions on sales and consumption, including:<br>• spatial availability, e.g., number, density or specific location of outlets<br>• temporal availability, e.g., hours of sale<br>• the environment of use, e.g., effects of neighbourhood regeneration, smoke-free zones<br><br>**Challenge question:** If sales or consumption were limited in one area, does your logic model show how individuals might counter the effects by moving to another area? |

# Cross-cutting policy area. Industry regulation

*Purpose:* Limit industry opportunity to influence policy and promote youth uptake

*Examples:*

- Monitor interference with information on health harms
- Limit involvement in the formation and implementation of local, national or international policy
- Disrupt coalitions across tobacco-alcohol-advertising industries
- Exclusion of psychoactive substances from trade agreements
- Prevent illicit sales / smuggling

*Links between tobacco and alcohol:*[1][2][3]

- Public health policy sectors can learn from each other, implementing policy more quickly and better.
- There is no current tobacco Responsibility Deal, so there is a divide between the respectability of the tobacco and alcohol industries.
- Transnational corporations employ common strategies, so it is likely that common rules for all industries would affect both tobacco and alcohol use. (including strategies to avoid government regulation)
- The tobacco industry is subject to stronger regulation that the alcohol industry - can the measures or perceptions applied to the tobacco industry also be applied to the alcohol industry?
- Countering the message by industry that policy should focus on heavy drinkers or ineffective individually-targeted information and educational approaches might create more political will for population-level policies that have the potential to affect both drinking and smoking.
- Disclosure of industry funding to e.g., alliances of trade unions, employees, and groups representing minorities opposing control policies.

---

[1] Moodie, R., et al. (2013). Profits and pandemics: prevention of harmful effects of tobacco, alcohol, and ultra-processed food and drink industries. Lancet 381(9867): 670-679.

[2] Jiang, N. and P. Ling (2013). Vested Interests in Addiction Research and Policy. Alliance between tobacco and alcohol industries to shape public policy. Addiction 108(5): 852-864.

[3] Casswell, S. (2013). Vested interests in addiction research and policy. Why do we not see the corporate interests of the alcohol industry as clearly as we see those of the tobacco industry? Addiction 108(4): 680-685.

# 1. Promotion: Social marketing

*Purpose:* To promote healthy choices and provide information on the links between product use and health (outside of the health system).

*How:* adverts, warnings and education via mass/social media, films, school/workplace initiatives

*Targets:* subjective norms, acceptable image, attitudes, awareness, knowledge, resilience, practices

*Examples:*

- focus on health outcomes with common tobacco and alcohol aetiology (e.g., frailty in old age)
- cancer risk (and physiological interactions between drinking & smoking)
- messages aimed at non-smokers/non-heavy users to emphasize benefits through positive messages

*Links between tobacco and alcohol:*

**From our pre-workshop survey**

- Decisions to improve health might be more broadly focused than a single behaviour. Providing healthy role-models could promote non-smoking/sensible drinking.
- Increased awareness of the joint role of tobacco and alcohol in cancer risk; framing alcohol in the same terms as tobacco - both as a carcinogen, but also a product that is not an ordinary commodity, produced and marketed by an 'unhealthy' industry might strengthen the message on alcohol.
- Many smokers attempt to cut down alcohol when they try to quit. But may also drink more.

**From our scoping review**

- School-based programmes aim to improve mental well-being, resilience, self-control and social/personal competence skills. The effects can be to:
    - lower tobacco and alcohol use among young people[4]
    - help them refuse offers, resist influences, correct misperceptions that use is normative[5].
    - make them less susceptible to influence by tobacco/alcohol advertising[6].

---

4 Hodder, R. K., et al. (2011). A school-based resilience intervention to decrease tobacco, alcohol and marijuana use in high school students. Bmc Public Health 11.

5 Botvin, G. J., & Griffin, K. W. (2007). School-based programmes to prevent alcohol, tobacco and other drug use. *International review of psychiatry*, *19*(6), 607-615.

6 Wills, T. A., et al. (2010). Good Self-Control Moderates the Effect of Mass Media on Adolescent Tobacco and Alcohol Use: Tests With Studies of Children and Adolescents. Health Psychology 29(5): 539-549.

## 2. Person: Measures to help individuals reduce use

*Purpose: Support individuals to quit or reduce their use of tobacco and/or alcohol products*

*How:*

- Identification and brief advice by care professionals, incl. promoting delivery and referral
- General support and information for individuals who are motivated to reduce use, incl. use of technology such as apps
- Specialist treatment services for individuals who need them to reduce use, incl. pharmaceuticals
- Support for mental health issues at all levels

*Links between tobacco and alcohol:*

**From our pre-workshop survey**

- Intervene in tobacco and alcohol at the same time vs. sequentially vs. separately? Certainly better to treat one than neither (i.e., counter the 'we can't intervene in smoking because alcohol is the problem' and vice versa).
- Many smokers attempt to cut down alcohol when they try to quit smoking. But others might replace the behaviour with increased alcohol use.
- Treatment for alcohol dependency might affect smoking cessation. Swapping addictions, e.g., people in recovery from alcohol or drugs often smoke more and eat a lot of sweets.
- Targeting mental health would likely affect both alcohol and tobacco consumption.

**From our scoping review**

- Brief alcohol interventions do not reduce smoking[7]. In non-dependent drinkers, changes in smoking were not found to be associated with changes in drinking[8].
- Smoking cessation treatment that incorporates brief alcohol intervention has been found to reduce alcohol use[9], but not in alcohol dependent smokers[10].
- In alcohol dependent individuals smoking abstinence reduced drinking[11]; smoking mildly increased relapse to drinking[12]; but nicotine can help with alcohol withdrawal symptoms[13].
- Alcohol use increased the risk of relapse to smoking[14]. But alcohol use reduced when smoking stopped and remained lower for six months post-cessation.

---

7 McCambridge, J. and R. J. Jenkins (2008). Do brief interventions which target alcohol consumption also reduce cigarette smoking? Systematic review and meta-analysis. Drug and Alcohol Dependence 96(3): 263-270.

8 Kahler CW, Borland R, Hyland A, et al. Quitting smoking and change in alcohol consumption in the International Tobacco Control (ITC) Four Country Survey. Drug and Alcohol Dependence. 2010 Jul;110(1-2):101-7.

9 Kahler, C. W., et al. (2008). Addressing Heavy Drinking in Smoking Cessation Treatment: A Randomized Clinical Trial. Journal of Consulting and Clinical Psychology 76(5): 852-862.

10 Cooney, N. L. et al. (2007) Concurrent Brief versus Intensive Smoking Intervention during Alcohol Dependence Treatment. *Psychology of addictive behaviors : journal of the Society of Psychologists in Addictive Behaviors* 21.4: 570–575.

11 Cooney, N. L., et al. (2015). Concurrent Alcohol and Tobacco Treatment: Effect on Daily Process Measures of Alcohol Relapse Risk. Journal of Consulting and Clinical Psychology 83(2): 346-358.

12 Dawson DA, Goldstein RB, Grant BF. Rates and correlates of relapse among individuals in remission from DSM-IV alcohol dependence: A 3-year follow-up. Alcoholism-Clinical and Experimental Research. 2007 Dec;31(12):2036-45.

13 Gulliver SB, Kamholz BW, Helstrom AW. Smoking cessation and alcohol abstinence: What do the data tell us? Alcohol Research & Health. 2006 2006;29(3):208-12.

14 Kalman, D., Kim, S., DiGirolamo, G., Smelson, D., & Ziedonis, D. (2010). Addressing tobacco use disorder in smokers in early remission from alcohol dependence: the case for integrating smoking cessation services in substance use disorder treatment programs. *Clinical Psychology Review*, *30*(1), 12–24.

## 3. Prescriptive: Regulation of industry advertising, marketing and sales

*Purpose:* Limit the opportunity and scope for tobacco and alcohol corporations to sell their products to consumers, particularly youth, via conventional advertising channels or via packaging and promotions

*Examples:*

- Restricting direct advertising (e.g., visual, online and print media, point-of-sale displays and sports advertising and sponsorship)
- Restricting indirect marketing (e.g., imagery in TV, films, product placement [c.f. BBFRC guidelines on sponsorship for TV] and social media)
- Especially through media accessed by young people (e.g., controls on time of day and context, social media and online marketing restrictions)
- Changes to packaging - regulated or voluntary addition / improvement of health warnings, information on contents, or removal of branding

*Links between tobacco and alcohol:*

**From our pre-workshop survey**

- More advertising restrictions on smoking via plain packaging may make further advertising restrictions on alcohol more likely as more cognitively available to policy makers.
- Alcohol-advertisements in sport expose children to messages concerning alcohol more than standard advertising programs, so adolescents may drink more, and thereby more likely to smoke.
- If the age-classification of films considered the frequency of smoking / drinking, perhaps this would limit the number of occurrences.
- Policing of online/social media marketing by alcohol and tobacco industries should have one set of clear rules for all – this might reduce both intake of tobacco/alcohol and rates of initiation.

**From our scoping review**

- In nicotine dependent, non-alcoholic smokers, imagery of social drinking was found to strengthen both cigarette and alcohol cravings. The same was found for imagery of smoking[15].
- Tobacco brand placement in the U.S. was limited in 1998, but this had little effect on (industry self-regulated) alcohol brand appearances, which tended to increase in youth-rated movies[16].
- Ads might link cigarette and alcohol sales, e.g., cigarette promotions featuring alcohol discounts or encouraging alcohol use[17].
- Preventing coalitions or co-ownership of tobacco, alcohol and advertising companies might prevent the industries sharing resources and lobbying power.
- Prevent alcohol/tobacco industry sponsorship of intergovernmental events, funding of educational initiatives, research, publications and sponsoring sporting and cultural events.[18]

---

15 Erblich, J., Montgomery, G. H., & Bovbjerg, D. H. (2009). Script-guided imagery of social drinking induces both alcohol and cigarette craving in a sample of nicotine-dependent smokers. *Addictive behaviors*, *34*(2), 164-170.

16 Bergamini, E., et al. (2013). Trends in Tobacco and Alcohol Brand Placements in Popular US Movies, 1996 Through 2009. Jama Pediatrics 167(7): 634-639.

17 Jiang, N., & Ling, P. M. (2011). Reinforcement of smoking and drinking: tobacco marketing strategies linked with alcohol in the United States. *American journal of public health*, *101*(10), 1942-1954.

18 See cross-cutting policy area on industry regulation.

## 4. Price: Fiscal policies, e.g., Taxation, minimum prices and levies

*Purpose:* Increase product price and therefore decrease affordability; increase fiscal pressure on corporations that manufacture tobacco and alcohol products; fund prevention activities and measures to reduce burden

*Examples:*

- Taxation (excise: by volume, unit or e.g. alcohol content; ad valorem: by proportion of the value)
- Minimum unit pricing (MUP)
- Polluter pays measures e.g. annual levy on tobacco and alcohol companies/retailers selling alcohol & tobacco (in proportion to market share or volume of sales)
- Fines for failures to meet compliance e.g. the illicit trade

*Links between tobacco and alcohol:*

**From our pre-workshop survey**

- Effects of taxation or MUP can differ in effects by product size, content or initial price and industry can modify the effects of taxation by changing profit margins on certain product types, e.g., to maintain ultra-cheap products
- An increase in the price of one product type might affect the purchase or perceived affordability of another (decreasing both smoking and drinking/inadvertently increasing consumption of another product type?)
- Increased price might reduce other discretionary spending, but reduced consumption would increase disposable income: how do people reallocate their budget?
- People could consume cheaper tobacco or alcohol, or consume less but in a way that doesn't reduce harm, e.g., smoking 'harder', or consume in different setting to reduce costs (c.f. place)
- Changing price affects consumption practices (e.g. place) and it is within these practices that an interplay between smoking and drinking resides.
- Make one unaffordable and either people will do more of the other or less of both, depending on personal circumstances and how ingrained behaviour is.

**From our scoping review**

- In the U.S., increased cigarette tax was linked to reduced alcohol consumption and binge drinking among smokers[19]. But only in male smokers. Stronger effect for hazardous drinkers, young adults, and adults with low income.
- Reductions in alcohol consumption associated with increased cigarette tax were seen for beer and spirits but not wine[20].
- Increased cigarette price raised binge drinking at young ages, and raised heavy drinking among young females. It reduced binge drinking among African Americans and Hispanics, and heavy drinking among Hispanics.[21]

---

[19] Young-Wolff, K. C., et al. (2014). Increased Cigarette Tax is Associated with Reductions in Alcohol Consumption in a Longitudinal US Sample. Alcoholism-Clinical and Experimental Research 38(1): 241-248.

[20] Krauss, M. J., et al. (2014). Effects of State Cigarette Excise Taxes and Smoke-Free Air Policies on State Per Capita Alcohol Consumption in the United States, 1980 to 2009. Alcoholism-Clinical and Experimental Research 38(10): 2630-2638.

[21] McLellan, D. L., et al. (2012). Unintended consequences of cigarette price changes for alcohol drinking behaviors across age groups: evidence from pooled cross sections. Substance Abuse Treatment Prevention and Policy.

## 5. Place: Restrictions on sales and consumption

*Purpose:* Limit opportunities for purchase and consumption of tobacco and alcohol products and support environments which encourage healthy behaviours

*Examples:*

- Use licensing to limit/restrict hours of sale, the number or density of outlets in certain areas (e.g. proximity relative to schools, overprovision)
- Age/proxy-purchase restrictions/ penalties
- Varied rules (e.g., on- vs. off-trade alcohol)
- Smoking bans in public places could be extended (e.g. smoke-free outside drinking venues)
- Increase availability of alternative choices (e.g., more non-alcohol centric venues for a night-out)

*Links between tobacco and alcohol:*

**From our pre-workshop survey**

- Restricting alcohol promotions (no happy hours) might reduce drinking and smoking in particular locations; but if drinking switches to home (e.g. preloading), second hand smoke exposure may rise.
- Smokers may stay inside non-smoking premises and drink more; smoke-free bars might lead smokers to increase home drinking (less regulated, larger measures, cheaper); smoke-free bars could make drinking more attractive to non-smokers.
- More evening venues without alcohol might reduce drinking and smoking as less pressure is felt e.g. by young people.
- Non-health policies (e.g. neighbourhood regeneration) might affect smoking and drinking in the streets (effects of boosting the night-time economy might not be limited to alcohol).
- Initiatives to tackle underage sales are likely to target alcohol and tobacco access by young people.

**From our scoping review**

- Alcohol and tobacco use increased for individuals closer to alcohol and tobacco retailers[22].
- In the U.S., smoke-free airs laws decreased consumption of beer and spirits but not wine[23]. Bans in restaurants and bars had more effect on beer and spirits, but increased demand for wine[24].
- In an international study, smoke-free policies caused small reductions in alcohol consumption by hazardous drinkers and in the frequency of alcohol consumption among heavy smokers[25].
- In England, smokers drank more than non-smokers before and after smoke-free policy[26]. The ban decreased drinking for smokers but increased drinking for non-smokers.
- In Scotland, smoke-free legislation was associated with reduced drinking behaviour in pubs and bars among moderate and heavy drinking smokers[27]. These moderate and heavy drinkers also reduced their pub attendance. But the smoke-free law did not increase drinking in the home.

---

22 Weitzman, E. R., et al. (2005). Youth smoking risk and community patterns of alcohol availability and control: a national multilevel study. Journal of Epidemiology and Community Health 59(12): 1065-1071.

23 Krauss, M. J., et al. (2014). Effects of State Cigarette Excise Taxes and Smoke-Free Air Policies on State Per Capita Alcohol Consumption in the United States, 1980 to 2009. Alcoholism-Clinical and Experimental Research 38(10): 2630-2638.

24 Gallet, C. A. and H. S. Eastman (2007). The impact of smoking bans on alcohol demand. Social Science Journal 44(4): 664-676.

25 Kasza, K. A., et al. (2012). Smoke-free bar policies and smokers' alcohol consumption: Findings from the International Tobacco Control Four Country Survey. Drug and Alcohol Dependence 126(1-2): 240-245

26 Orbell, S., et al. (2009). Social-Cognitive Beliefs, Alcohol, and Tobacco Use: A Prospective Community Study of Change Following a Ban on Smoking in Public Places. Health Psychology 28(6): 753-761.

27 McKee, S. A., et al. (2009). Longitudinal evaluation of smoke-free Scotland on pub and home drinking behavior: Findings from the International Tobacco Control Policy Evaluation Project. Nicotine & Tobacco Research 11(6): 619-626.
