# Supplementary material for: Conceptualising changes to tobacco and alcohol policy as affecting a single interlinked system
Source: BMC Public Health. 2021 Jan 4;21:17. doi: 10.1186/s12889-020-10000-3 (PMC7783976; doi:10.1186/s12889-020-10000-3)
Supplement: Supplementary file 3 — Additional file 3. [file 12889_2020_10000_MOESM3_ESM.pdf]

Tobacco and Alcohol Policy Modelling - developing a common policy modelling framework

Facilitator guidance

Thanks for agreeing to be a facilitator at the workshop. Your role is to support the two group sessions and to provide feedback to the workshop plenary at the end of the day.

You will be provided with
Also given to participants:<br>- Timetable of the day<br>- Instructions on 'what is a logic model' and 'how we're going to make one'<br>- One page briefs on our 5 policy areas<br>- Notebook and pen<br><br>Only for facilitators:<br>- Pro forma for recording information about elements of the logic model (see example in Table 1)<br>- Suggested prompt questions to help with logic model development<br>- Flipchart paper and pens

General points
Our data collection relies on the notes taken by facilitators and participants.<br><br>Throughout, please encourage participants to jot down their own thoughts in their notebooks and to be respectful of their co-participants.<br><br>Please ask participants to describe why they are making a particular statement and what assumptions they are making.

Session 1 (1130-1300)

Participants will be asked to self-select into breakout groups. Each group will have a facilitator and will be focused on one of our 5 policy areas. Ideally, the discussions in Session 1 shouldn't be influenced by considerations of available evidence – you may need to steer discussion away from this.

-- IN THE FIRST 30 MINUTES --

1. Shout-out if you don't have 3-5 participants with a mix of tobacco/alcohol, research/policy expertise – we'll try to re-arrange.

2. Tell your group that:
   - to fill-in the pro forma you will ask them for the rationale and assumptions behind each part of the logic model.
   - they should take notes in their notebooks, detailing their thought process as the logic model takes shape.

3. Discuss what a logic model is – looking at the example provided.
   - building the logic model is just a guide to thinking and discussion.
   - it is attempting to describe the real world so all important elements should be included even where no data is available.

4. Get your group to read the policy brief and then think of a specific policy option to be their initial focus. Prompt them to try to reflect what is happening or might happen in the UK.

5. Ask your group to write a list of what they might want to include, considering
   - outcomes for smoking and drinking, e.g., rates of initiation, use, attempting to cut-down, relapse.
   - outcomes other than smoking and drinking are allowed, if part of a feedback that is too complicated to describe.
   - agents in the system, e.g., industry, government, individuals.
   - key factors, concepts or variables, e.g., from economic or psychological models of behaviour.

-- IN THE LAST 60 MINUTES --

6. Get your group to begin their logic model on flipchart paper - using their list as a starting point. Suggest that participants to first write their outcomes at the top of the paper.

7. Get a good discussion going – see our suggested prompt questions.

Session 2 (1345-1500)

During lunch, each group's logic model will have been displayed on easels around the room. You should stand by your group's logic model to explain it to others whilst groups rotate around the room.

1. Try to take your own notes as you'll need to give a 3 minute summary of the comments you receive.

2. In the session, you'll see 4 groups, with around 15 minutes per group.

3. When a group arrives, briefly introduce your logic model and remind participants to make their own notes of their thoughts/comments.

4. First ask each group to critique your model, e.g., participants may suggest additions or amendments, express agreement or disagreement with model components or assumptions.

5. Second, ask for views on what data is available to populate the model and any new research that would be required to action the model.

Plenary session (1505-1600)

We'll put up a photo of your logic model on the projector screen while you give your summary.

1. In your summary, try to spend a minute each on:
   - an overview of your logic model.
   - the critique you received in session 2.
   - how current and potential future evidence might be used.

2. In the following open discussion among participants and facilitators, please take notes of what is said.

Suggested prompt questions to help with logic model development

OUTCOMES

1. Do any of the following aspects of behaviour need to be distinguished in your model?
   - the contexts in which behaviours occur
   - impact duration.
   - attempts to change or success at changing.
   - prevention of uptake versus cessation versus reduction.

SOCIETAL VARIATION / INEQUALITIES

2. Have you identified the components that are likely to explain variation in policy effects according to:
   - age and sex?
   - socio-economic and cultural factors? (incl. economic activity, income, ethnicity, family situation)
   - state of health? (incl. clinical diagnoses, general fitness and wellbeing)

3. Have you been explicit about which groups you are talking about in terms of behaviour, e.g., adult smokers, not drinkers; drinkers, not smokers; smokers and drinkers; and similarly for youth untried/have tried/occasional/regular).

CONTEXTS

4. Have you described the contexts in which people are smoking/drinking/both/neither?

MODEL BREADTH

5. Are there any other (positive or negative) consequences of the policy option or its intermediate outcomes?

MODEL COMPLEXITY

6. Could there be any factors which explain two outcomes?

7. Are there any other possible links between factors? (with the aim of establishing whether there are any feedback loops)

8. Might a third party act to reduce the impact? (with the aim of establishing how industry might circumvent policy actions)

Table 1: Example pro forma to help note-taking during session 1

Is it a link (L) or a node (N)?	What is the name of the Link/Node?	How does it relate to the policy option / Why are we including it in the logic model?	Areas of agreement or disagreement?	What assumptions have been made about the node or link?
N	Cake eaten in workplace	Any cake eaten in the workplace will have an effect on dietary intakes and social expectations.	Everyone agreed inclusion but we unsure what qualifies as cake? E.g. are scones cake?	Cake eaten in the lunch break but still in the office counts as cake eaten in the workplace.
L	Cake eaten in workplace - Daily fat intake	The consumption of cake will increase fat intake.	What if office workers bring low-fat cakes to the office?	
